# Supplementary material for: Third COVID-19 vaccine dose boosts neutralizing antibodies in poor responders
Source: Commun Med (Lond). 2022 Jul 11;2:85. doi: 10.1038/s43856-022-00151-2 (PMC9273613; doi:10.1038/s43856-022-00151-2)
Supplement: Supplementary file 2 — Description of Additional Supplementary Files [file 43856_2022_151_MOESM2_ESM.pdf]

Description of Additional Supplementary Files

File Name: Supplementary Data

Description: De-identified raw data is recorded in density units and available upon request. Source data refers to percent neutralization calculations generated from raw data and is included with this manuscript. Data used to generate Figures 2, 3, and Supplementary Figure S1 are available in the file 'Supplementary Data'.
