# Supplementary material for: Third COVID-19 vaccine dose boosts neutralizing antibodies in poor responders
Source: Commun Med (Lond). 2022 Jul 11;2:85. doi: 10.1038/s43856-022-00151-2 (PMC9273613; doi:10.1038/s43856-022-00151-2)
Supplement: Supplementary file 3 — Supplementary Materials [file 43856_2022_151_MOESM3_ESM.pdf]

Supplementary Materials

This material has been provided to give readers additional information about the work.

Supplement to: Douglas F. Lake, Alexa J. Roeder, et. Al. Third COVID-19 Vaccine Dose Boosts Neutralizing Antibodies in Poor Responders

Supplementary Figure S1 – Regression analysis of LFA density values and microneutralization IC_{50} values.

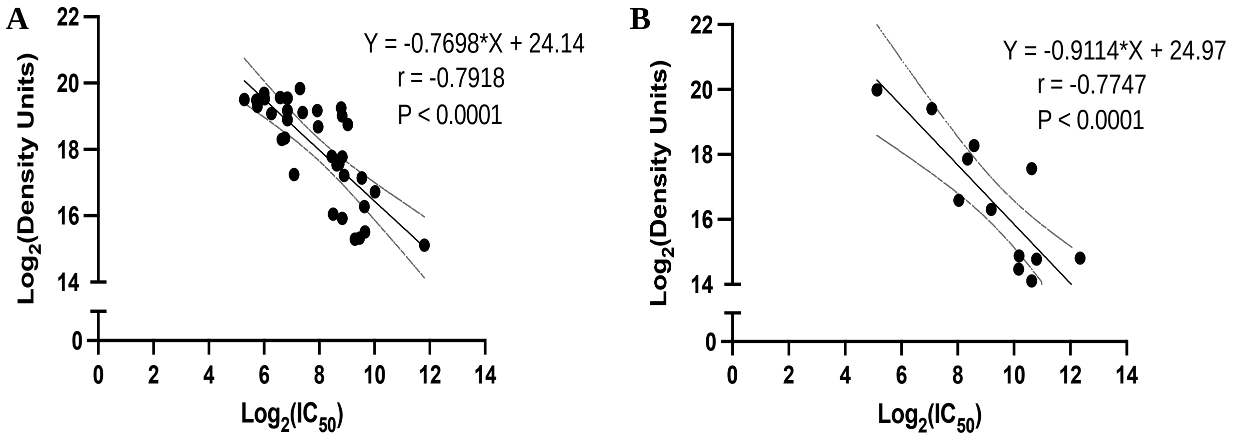

Supplementary Figure S1. Comparison between LFA density units and IC_{50} values obtained using **A)** convalescent sera isolated from 36 recovered COVID-19 patients and **B)** vaccinated sera from 13 individuals. Neutralization was tested on LFA using 6 µL serum and a dilution series was performed by authentic SARS-CoV-2 FRNT assay to obtain IC_{50} values. To calculate IC_{50}, data were analyzed in GraphPad Prism 9.0 using methods described by Ferrara and Temperton. Density values and IC_{50} values were Log_2-transformed and analyzed using a simple linear regression and nonparametric Spearman correlation with two-tailed P value and a 95% confidence interval (CI). Regression analysis with 95% CI boundaries is indicated in solid black and grey dotted lines. Spearman's rho and two-tailed P value are labeled.

Supplementary Figure S2 – NAb LFA Tests with Density Values, IC50s, NAb Titers, and Percent Neutralization

	VAC-93	VAC-81	VAC-77	VAC-82	VAC-74	VAC-99
**TLD**	1,007,231	765,297	477,787	295,032	156,481	14,212
**IC_{50}**	43.8	62.6	115.4	215.9	492.2	11,103.9
**NAb Titer**	≤ 40	≥40, <80	≥80, <160	≥160, <320	≥320, >640	≥640
**% Neut**	0	19	49	69	84	99

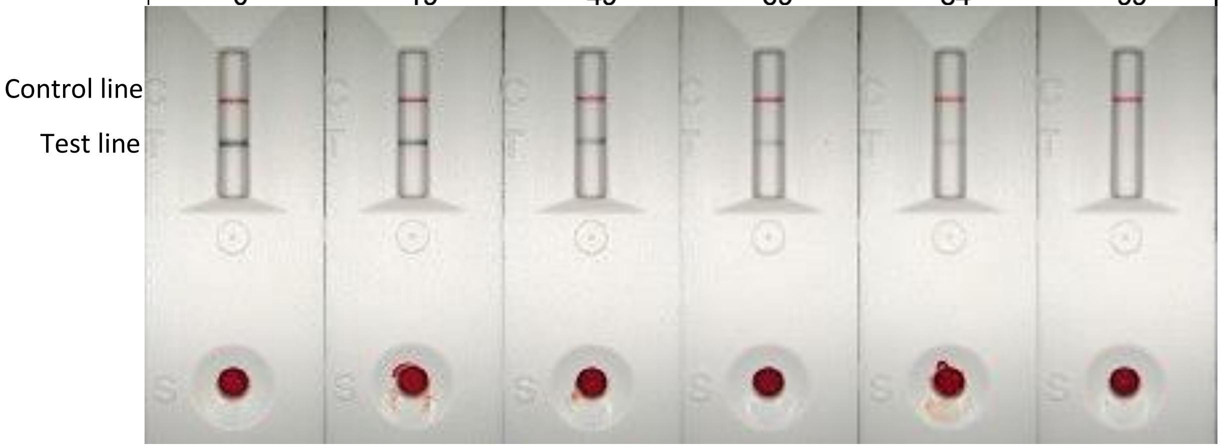

Supplementary Figure S2. LFA densitometer images with corresponding Test Line Density (TLD), IC_{50} value, reciprocal NAb titer, and percent neutralization. TLD is reported as unmodified, discrete values. IC_{50} values are derived from the regression equation shown in Supplementary **Figure S1**. Reciprocal NAb titer is derived from IC_{50}, such that titer is equal to the last dilution factor (DF) ($DF = 20*2^{-n}$) for which IC50 is ≥ the lower threshold range for a given titer. For example, IC_{50}=43.8 is classified as an NAb titer of 40. Further, a hypothetical IC_{50}=158 would classify as NAb titer ≥80 and <160. Percent neutralization was calculated using the equation: 1-(TLD/LoD) when LoD=942,481 as described in **Table 1** legend.
